# Exploring clonal hematopoiesis and its impact on aging, cancer, and patient care

**DOI:** 10.18632/aging.205404

**Published:** 2023-12-20

**Authors:** Julieta Elena Rodriguez, Jean Baptiste Micol, Capucine Baldini

**Affiliations:** 1 Drug Development Department, Gustave Roussy, France; 2 Department of Hematology, Gustave Roussy, France.

**Keywords:** clonal hematopoiesis, aging, solid tumors

Clonal hematopoiesis (CH) is a term that refers to the presence in blood cells of hematologic malignancy-associated somatic mutations without fulfilling the diagnostic criteria of hematologic disease [1]. Emerging evidence suggests that CH is a consequence of an expansion of cells harboring initiating driver mutations, potentially linked to the aging hematopoietic system [2]. While these detectable somatic mutations are rare in individuals under 40 years old, they become increasingly prevalent in the elderly population, a term called age-related clonal hematopoiesis (ARCH), reaching up to 18.4% in those aged 90 years or older [1].

**Figure 1:**
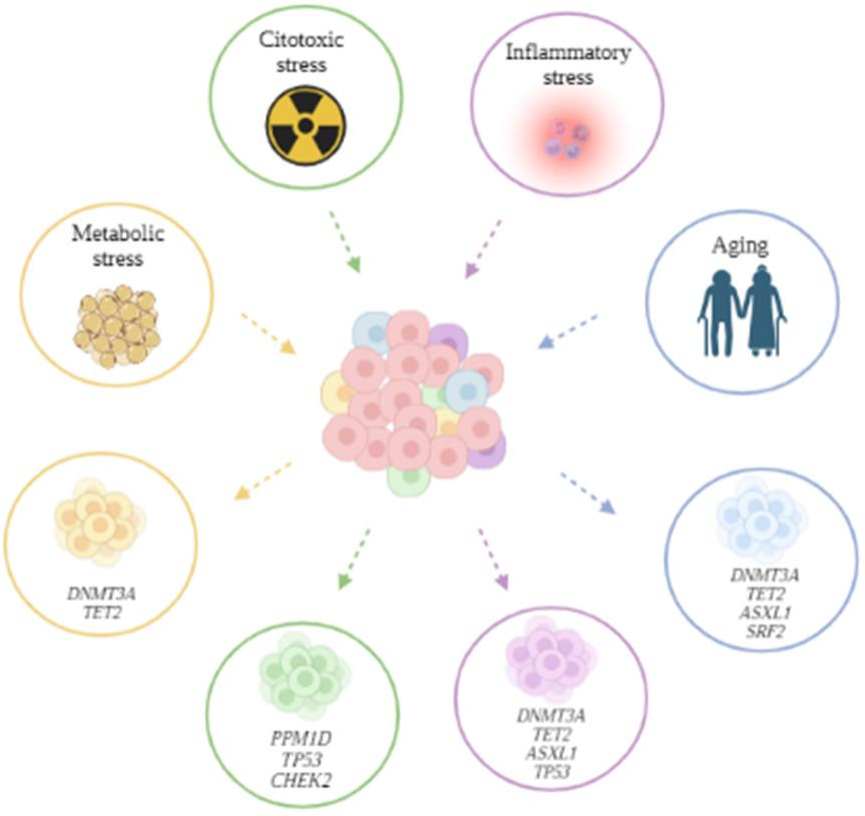
Stressors and CH clones.

Aging itself is a significant stressor associated with CH, particularly in individuals over 70 years old. *DNMT3A*, *TET2*, and *ASXL1* [2] mutations are more common with advancing age. Mechanistically, age-related clonal expansion is believed to overlap with inflammatory stimuli, with increased production of pro-inflammatory cytokines contributing to the expansion of *DNMT3A* and *TET2* CH clones. Additionally, aged cells exhibit altered DNA and histone methylation, leading to self-renewal signatures activation and differentiation program silencing. Importantly, prior exposure to anticancer therapies has been linked to the expansion of specific CH clones. Mutations in DNA damage response genes, including *TP53*, *PPM1D*, and *CHEK2*, are strongly associated with such stress [3]. Understanding the influence of prior treatments on CH dynamics is crucial for comprehending the intricate interplay between therapy, aging, and cancer (Figure 1).

Recent evidence also indicates that CH may play a role in solid tumors, such as an increased risk of incident lung cancer [4]. While initial studies associated CH mutations with worse survival outcomes [5], newer findings suggest that solid tumor patients with CH may experience longer survival [6]. However, the underlying mechanisms behind this relationship remain to be elucidated.

The actual influence of CH on patients with solid tumors remains an area under investigation. Advancing age has been associated with a higher incidence of both cancer and cardiovascular ailments. Studies have demonstrated that individuals harboring mutations in DNMT3A, TET2, and ASXL1 genes face a 1.7 to 2.0-fold increased risk of developing coronary heart disease compared to those without such genetic variations [2]. Moreover, experiments conducted on murine models of atherosclerosis revealed that the depletion of Tet2 function in hematopoietic cells expedited the progression of atherogenesis. Recent research suggests a higher prevalence of CH in patients with solid cancer, with approximately 30% exhibiting CH mutations in their bloodstream [7]. As the use of genetic sequencing in oncology decision-making rises, the inadvertent identification of CH during genomic analysis becomes more likely. Extensive investigations are imperative to fully grasp the implications of CH in the realm of solid tumors and its potential impact on patient management.

Previous exposure to various chemotherapeutic or radiotherapeutic agents may promote the development of specific CH clones, as observed in the cases of *TP53* or *CHEK2* mutations. Distinguishing whether these alterations involve hematopoietic clones or mutations specific to the solid tumor is of particular interest [8], especially when these abnormalities are identified in an attempt to discover potential therapeutic targets to guide further treatments in patients with solid tumors and limited therapeutic options.

In conclusion, as we continue to advance in genetic sequencing and oncological decision-making, the possibility of unintentionally identifying CH during genomic analysis becomes more likely. Therefore, investigating CH in the context of solid tumors is a complex and evolving field, holding significant promise for future therapeutic strategies and personalized patient management. Further research and exploration of these mechanisms are essential to unlock the full potential of CH as a predictive and prognostic marker in the field of oncology.
